# Impact of carpal tunnel syndrome on the expectant woman's life

**DOI:** 10.1186/1447-056X-11-1

**Published:** 2012-01-30

**Authors:** Zatel I Rozali, Faiz M Noorman, Prisca K De Cruz, Yam K Feng, Halimatun WA Razab, Jamari Sapuan, Rajesh Singh, Faizal M Sikkandar

**Affiliations:** 1 Department of Orthopaedic, Faculty of Medicine, Universiti Kebangsaan Malaysia Medical Centre, Jalan Yaacob Latiff, Bandar Tun Razak, 56000 Cheras, Kuala Lumpur, Malaysia

**Keywords:** Carpal Tunnel Syndrome, third trimester pregnancy, prevalence, impact, severity, functional

## Abstract

**Introduction:**

Carpal Tunnel Syndrome is known to be a common complication during pregnancy especially during the third trimester.

**Aim:**

This article focuses on its impact to the third trimester pregnant mothers with CTS.

**Methods:**

Third trimester pregnant mothers with no other known risk factors for CTS, were interviewed and examined for a clinical diagnosis of CTS. The severity of CTS was assessed by means of symptoms severity and functionality using the Boston Carpal Tunnel Questionnaire.

**Results:**

Out of 333 third trimester pregnant mothers, 82 (24.6%) were clinically diagnosed with CTS. Malay race was found to have significant correlation with the diagnosis of CTS (p = 0.024) and are two times more likely to get CTS during pregnancy (OR = 2.26) compare to the non-Malays. Bilateral CTS was two times higher (n = 58, 63.4%) than unilateral cases (n = 30, 36.6%), however no significant correlation between the two was found with severity (p = 0.284) or functional (p = 0.906). The commonest complaint was numbness/tingling during day time (n = 63, 76.8%). Majority of the CTS cases were mild (n = 66, 80.5%) and approximately one third (n = 28, 34.1%) had affected hand functions. All symptoms related to pain was found to have significant correlation with severity (p = 0.00, OR = 12.23) and function (p = 0.005, OR = 5.01), whereas numbness and tingling does not (Severity, p = 0.843, function, p = 0.632).

**Conclusion:**

This study shows that even though CTS in third trimester pregnancy is prevalent, generally it would be mild. However, function can still be affected especially if patients complain of pain.

## Introduction

CTS occurs when the median nerve is entrapped within the Carpal Tunnel causing sensations of pain, numbness and tingling at the median nerve distribution in the hand and could extend up to the arms. Previous studies had found that CTS was more prevalent in the female population. It was postulated because morphologically, females are more prone to CTS compare to male [1]. CTS were first linked with pregnancy in 1957 by Wallace and Cook where they describe two cases of CTS in pregnancy and did surgical decompression [2]. The paper focuses on "pressure as the cause of such symptoms". Subsequent studies made and more theories of CTS in pregnancy were proposed. As more studies begin to address CTS in pregnancy, there was still no equivocal estimate of how common it is in pregnancy and how severe it could be. A study by Voitk et al. found that 24.9% of pregnant mothers had hand symptoms in the median nerve distribution, where 51% found the symptoms annoying, disturbed functions in 42%, 29% claimed that it interferes with sleep and 4% found that it was intolerable [3]. Voitk et al. also recorded that only 49% mentioned the symptoms to their doctor in which only 16% were treated or given advice.

Luckily many study found that the timing of the first pain of CTS occurs during pregnancy was the highest during the third trimester [4,5] rather than facing the pain throughout the pregnancy. However there is not much literature to assess how much CTS actually affects their daily life as pregnant mothers particularly during in that third trimester. CTS might be the least of problem that these woman had to face during pregnancy in which most of them experienced resolution[6,7] or improvements after delivery,[8] but the fact is, there are no real data to address this problem. Therefore this study focuses on how much CTS actually affects a mother's life during the third trimester pregnancy.

## Methods

### Study Design

This study is a prospective cross sectional study involving 333 third trimester pregnant mothers from UKMMC Obstetric clinic.

### Subjects and Settings

It was conducted from October 2010 till April 2011. Ethical approval by UKM Ethical Committee was obtained for this study. The pregnant mothers were selected at the UKMMC Obstetric Clinic during their antenatal follow up. Only pregnant mothers who were in their third trimester were approached. After taking consent, they were asked regarding exclusion criteria namely Gestational Diabetes Mellitus, Hypertension, athropathies, thyroid disease, trauma at the hand or wrist and pre-existing or history eclampsia or pre-eclampsia, previous diagnosis of CTS and recurrent CTS. Clinical diagnosis of CTS was made from history and physical examination. Pregnant mothers with normal findings from physical examination and history were grouped as non-CTS. For the CTS group, subjects with onset of symptoms during first and second trimester were excluded. Subjects with distribution of symptoms other than median nerve distribution, abnormal lumps or bumps at the hand or wrist, or positive for higher nerve compression were also excluded. This was done to ensure that the CTS group in our study is not because of other pathology in order to study the true nature of CTS in pregnancy.

### Instruments

The history taking was standardized using a questionnaire consisting or three main parts,

(a) Demographic data, age, parity, registration number, and gestational weeks;

(b) Diagnosis of CTS. The pregnant mothers were first asked whether they had been diagnosed with CTS or not. Six-item Carpal Tunnel Syndrome Scale (CTS-6) questions were used for the diagnosis of CTS [9]. In order to refined the scale, another item was added to the CTS 6 which was "shaking the hand relieves the hand symptoms". One or more symptoms in the CTS-6 are enough to suggest CTS and they will be asked to map the site of the symptoms.

(c) Underreported CTS. The pregnant mothers were also asked whether or not they had mentioned their symptoms to the doctors and whether treatment was given for those who mentioned.

The severity of CTS was evaluated using the Boston Carpal Tunnel Questionnaire (BCTQ) [10]. The BCTQ consist of two measurements of disease's severity, the symptoms severity and functional status. The BCTQ is also the main instrument used with the aim of studying the impact of CTS in pregnant mothers.

### Statistical Analysis

The data was analysed using the Statistical Package for Social Sciences (SPSS) 13. In order to estimate frequency (in percentage), standard descriptive statistical methods were used on all variables except for the age which was a continuous data therefore expressed in mean and standard deviations. Relationships among categorical variables including the BCTQ symptoms severity and functional severity were determined using Chi-Square (X^2^) test. The BCTQ scores for symptoms severity and functional status was also summed up into group totals as proposed by Store et.al [11] as it was found to be the best way to present the data. The score totals used for symptoms severity was categorized into asymptomatic (11), mild (12-22), moderate (23-33), severe (34-44) and very severe (45-55). Functions scores were grouped into asymptomatic (8), mild (9-16), moderate (17-24), severe (25-32) and very severe (33-40) [10]. For associations that were found to be significant, odds ratio (OR) and 95% confidence interval was also calculated. P value of less than 0.05 was considered as statistically significant.

## Results

In total, 333 third trimester pregnant mothers, with age ranged between 17-45 years old had agreed to participate in this study. There are multiracial involvement amongst the respondents consisting of the Malays (n = 263, 79%), and non Malays; Chinese (n = 54, 16.2%), Indians (n = 8, 2,4%) and other races (n = 8, 2.4%). The National Statistics Department had revealed that Malaysia had a population of 25.7 million of which 24 million Malaysians (93.4%) and 1.7 million were non-Malaysians (6.6%). Among the non-Malays, Indians made up 1.8 million (7.5%), Chinese 6.1 million (25.4%), other races 300,000 (1.3%) [12]. Therefore, the distribution of races in our study might not be exactly the same with the racial distribution in Malaysia, however it is similar in which most of Malaysians are Malays followed by Chinese, Indians and others.

82 pregnant mothers (24.6%) were clinically diagnosed to have CTS where 72 (87%) of them were Malays, and 10 (12%) were non-Malay which comprise of Chinese (n = 9, 11%) and Indians (n = 1, 2.4%). The correlation of the Malay race with CTS in this study was found to be significant (p = 0.024) and the risk for Malay pregnant mothers to get CTS was twice higher than the non-malays (OR = 2.26). Other variables such as gravida, parity, and age have no significant correlation with CTS.

The cases of bilateral CTS in pregnancy (n = 58, 63.4%) were almost two times more than the unilateral cases (n = 30, 36.6%). However, whether it is bilateral or unilateral CTS, there was no significant correlation with the severity of CTS (p = 0.284) or functional (p = 0.906). From the physical examination, 11% had affected 2 point discrimination and 8.5% had weakness of the abductor pollicis brevis muscle.

**Figure 1:**
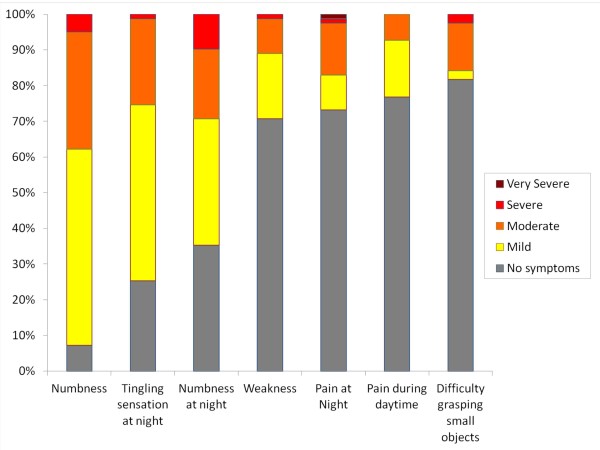
**Symptoms severity by each complaints**.

The most common presenting symptom from the CTS-6 was numbness and tingling during daytime (n = 63, 76.8%), followed by numbness and tingling at night, (n = 59, 72%), relief by shaking (n = 43, 52.4%), waking up due to numbness (n = 27, 32.9%), pain during day and night time (both n = 20, 24.4%), and the least common symptom was waking up due to pain (n = 14, 17.1%). Similarly, the most common complaint from the BCTQ symptom severity scale (Figure 1) was also numbness (n = 76, 92.7%), where 45 (54.9%) had mild numbness, 27 (32.9%) moderate numbness and 4 (4.9%) had severe numbness. The other two common complaints were tingling sensation (n = 62, 75%) and numbness at night (n = 53, 64.6%). Even though pain was the least common complaint for CTS in pregnancy it was found that complains of any symptoms related to pain have significant correlation with severity (p = 0.00, OR = 12.23) and function (p = 0.005, OR = 5.01), whereas numbness and tingling did not (Severity, p = 0.843, function, p = 0.632). Among those who complained of pain, numbness or tingling at night, 13 (15.9%) woke up because of pain and 27 (32.9%) woke up because of numbness or tingling sensation. However, both complaint of waking up due to pain or waking up due to numbness had significant correlation with impaired function (p = 0.001, p = 0.000 respectively).

**Figure 2:**
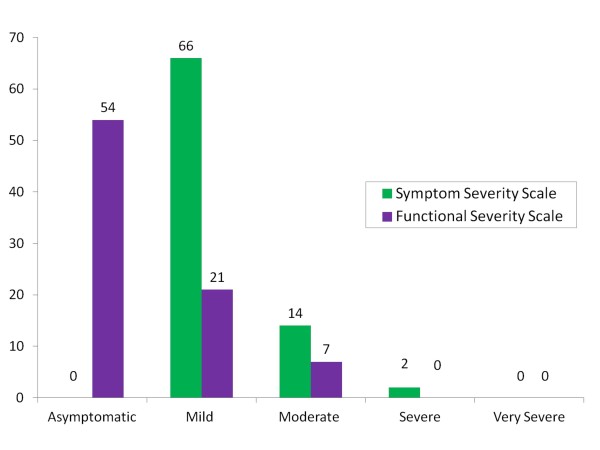
**Frequency of symptoms and functional severity according to group totals**.

**Figure 3:**
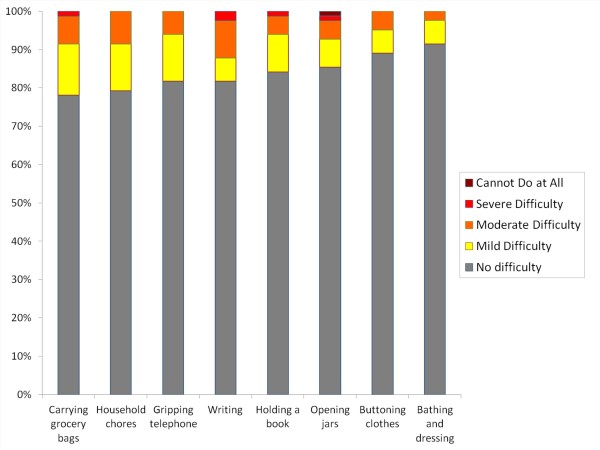
**Functional severity by each task**.

As a whole, summation of BCTQ-Symptoms severity scale (Figure 2) revealed that, most of the CTS cases in third trimester pregnancy was mild (n = 13, 80.5%), 7 (17.1%) had moderate CTS and only 1 respondent (2.4%) had the severe form of CTS. Whereas, the BCTQ-Functional scores (Figure 2) found that 28 (34.1%) of symptomatic pregnant mothers had functional difficulties, where 21 (25.6%) had mild and 7 (8.5%) had moderate functional difficulties. There were no respondents' scores higher than moderate functional score. The function from the BCTQ-functional score according to task is shown in Figure 3. The task that was commonly affected were carrying grocery bag (n = 18, 23%), followed by doing household chores (n = 17, 20.7%), gripping telephone (n = 15, 18.3%) and writing (n = 15, 18.3%), holding a book (n = 13, 15.9%), opening a jar (n = 12, 14.6%), buttoning clothes (n = 9, 14.6%) and bathing and dressing (n = 7, 9%).

Despite all this, only 21 (25.6%) of the symptomatic pregnant mothers mentioned their problems to the doctor in which only 2 (9.5%) receive treatments, such as vitamins, painkillers, calcium supplements and advice to drink plenty of milk. There was a significant correlation between the number of pregnant mothers who mention their symptoms to the doctors with the severity (p = 0.013, OR = 4.08) and function (p = 0.041, OR = 2.84). In addition to that, it was also observed that none of the pregnant mothers knew about CTS except for those who work in the clinical health setting such as doctors and nurses. However no objective data were taken to support this. But this fact further strengthens the impression that CTS is still not well known to the community, not even to the high risk group of pregnant mothers.

## Discussion

Recognizing the prevalence of CTS in pregnancy gives an overview on how common and important it is in the community. The prevalence of CTS in pregnancy varies from the previous researches. Padua et.al and Pazzaglia [13] et.al recorded more than 50% incidence of CTS in pregnancy, Voitk et al. recorded that it was 34% while Stolp et al. found that it was less than 1%. In our study, the prevalence was 24.6% or one fourth of uncomplicated third trimester pregnancy. We choose to study specifically the woman in the third trimester as previous study found that most CTS during pregnancy occur during the 3^rd ^trimester [4,5]. Compared to other studies, this study also excludes CTS in Diabetic patients in which was a known risk factor for CTS. Other diseases which are common in pregnancy, (thyroidism and hypertension) and higher nerve compression were also excluded. Therefore, it can be concluded that the small number of CTS patients was because the respondents selected were only those who had pure idiopathic CTS during pregnancy. This is an important criterion as only the pure idiopathic CTS in pregnancy respondents were indentified. This allows us to get the appreciation of the true nature of idiopathic CTS in pregnancy. Previously there was no study that found CTS had significant correlation with ethnicity. Therefore, the finding that CTS was twice as common in the Malay is an important finding for the Malaysian population. The pathology and reasons however, was unknown as this was the first finding documented in literature.

The incidence for unilateral and bilateral CTS in pregnancy was almost the same, with bilateral cases being higher than unilateral cases. Whether the CTS were unilateral or bilateral, it has no significant correlations with increasing of severity or impairment of function. This was the same with one of the studies by Padua et.al on CTS in general population where bilateral cases was also higher but no significant correlation with severity [8].

In this study BCTQ was used, as it is a standardised, patient-based outcome measure of symptom severity and functional status in patients with carpal tunnel syndrome. The use of BCTQ in this study was also preferred as it was found to be valid, reliable, responsive and it is an acceptable instrument to assess CTS according to a review on BCTQ [14]. In the present study, 80.5% of pregnant mothers were found to have mild symptoms of CTS and smaller number was found to have moderate CTS. Therefore even though CTS in pregnancy could occur in one fourth of pregnant mothers with no other risk factor for CTS, the severity will not extend beyond moderate. However, considering that pregnant mothers would also experienced other difficulties such as back pain, abdominal pain, or leg swelling, such disturbances could be a significant stressor to the mother.

The result in Figure 1 shows that the nature of CTS in third trimester pregnant mothers revolves more on numbness and tingling. Numbness or paraesthesia was also found to be the most common symptoms of CTS in pregnancy and CTS in general [15]. On the other hand, even though numbness and tingling was more common than all the other complaint, it was not as troubling as pain. Voitk et.al documented that pain was a prominent feature in 67% of symptomatic pregnant mothers. In the present study, all of the symptoms in CTS-6 regarding pain did not have any significant correlation to the symptoms severity, but it was found to have a significant correlation with impairment of function. Whereas for numbness, only waking up due to numbness had significant correlation with impairment of function. This signifies that once a patient complains of pain, regardless of severity, the function would usually become impaired. Pregnant mothers who complain of pain also have 12 times higher risk getting moderate to severe CTS. Compare to respondents who complains of numbness only, not many had functional difficulty unless it was severe enough to wake them up at night. Another way to interpret this is that both waking up at night because of the symptoms, whether its pain or numbness, have significant correlation with functionality. Therefore, mothers with sleep difficulty find CTS more disturbing during the day than those whose sleep not affected. This can be a good indicator for future studies on impact of CTS particularly during pregnancy.

Even though more than 80% of CTS cases in pregnancy were found to be mild, one third of the pregnant mothers with CTS had some degree of functional difficulties. The most affected task involves doing heavy work such as carrying grocery bag and household chores. Since some pregnant mothers who are in their third trimester usually avoid hard work, the functional difficulties may not be so prominent. This could also be the reason why the number of pregnant mothers that mention their symptoms to their doctors was only 26%. However what would be more of a concern was that among those who mention the symptoms to their doctors, less than 10% were given treatment. Not only that, most of the treatment offered was not related to treating CTS such as giving vitamins, calcium supplements and advice to drink milk. In one previous study, it shows that CTS symptoms were being underreported by patients as only 46% of symptomatic patients complain about their hand symptoms to practitioners. Out of that, only 35% were given treatment [3]. The lower incidence in the present study was probably because of a different country's health role, also socioeconomic and education level factors in subjects. In contrast, this study had proven that CTS, even though only mild in severity, could impairs a mother's in the crucial last trimester and doctors should at least be able to acknowledge this fact to offer some relief to the pregnant mothers.

## Conclusions

CTS in pregnancy should be given more attention since it is common in the Malaysian population especially in the Malays. Though the extent of symptoms severity may not be worrisome to the doctors, it could impair the daily functions of these pregnant mothers. The symptoms of pain should be given a greater consideration as it was found to commonly affect hand functions. Doctors should at least be able to identify, educate and offer conservative measures if needed, to mothers with CTS during pregnancy.

### Summary of Implications for GP

CTS is common in the third trimester pregnancy. General practitioners should be on the lookout for CTS in pregnancy and ask the patient about it. Most commonly, it is under reported as majority of the cases are mild and would not disturb functions. In the case of pregnancy, the classical presentation of nocturnal paraesthesia is still the hallmark of CTS, however, the symptoms of pain may more likely to affect the mother's daily life routines. Proper education and reassurance should be given to the patient and referral should be given in case of severe CTS.

## List of Abbreviations

CTS: Carpal Tunnel Syndrome; UKM: Universiti Kebangsaan Malaysia; UKMMC: Universiti Kebangsaan Malaysia Medical Centre; CTS-6: Six item Carpal Tunnel Syndrome [atroshi]; BCTQ: Boston's Carpal Tunnel Questionnaire; X^2 ^= Chi Square; OR: Odds ratio

## Competing interests

The authors declare that they have no competing interests.

## Authors' contributions

ZIR, YKF, WNH, PKDC, and MFN have the same weightage of contributions in carrying out the study from the literature reviews, proposals, interviewing the subjects, analyze the data, and preparing the manuscript. ZIR is the chief writer of this manuscript assisted by all the other authors above and MFN coordinated the study in general. RS and JS supervise all the works, made big decisions for the study, provides assistance in areas of administrations and give comments and ideas to improve the study in general. MFN assists in the communication between the statistician and ethical committee and participated during the interviews with the subjects. All the authors read and approve the final manuscript.
